# Natural infection of the sand fly *Phlebotomus kazeruni *by *Trypanosoma *species in Pakistan

**DOI:** 10.1186/1756-3305-3-10

**Published:** 2010-02-25

**Authors:** Hirotomo Kato, Hiroshi Uezato, Hiroshi Sato, Abdul M Bhutto, Farooq R Soomro, Javed H Baloch, Hiroyuki Iwata, Yoshihisa Hashiguchi

**Affiliations:** 1 Department of Veterinary Hygiene, Faculty of Agriculture, Yamaguchi University, 1677-1 Yoshida, Yamaguchi 753-8515, Japan; 2 Department of Dermatology, Faculty of Medicine, University of the Ryukyus, 207 Uehara, Nishihara-cho, Okinawa 903-0215, Japan; 3 Department of Veterinary Parasitology, Faculty of Agriculture, Yamaguchi University, 1677-1 Yoshida, Yamaguchi 753-8515, Japan; 4 Department of Dermatology, Chandka Medical College, Doctors Colony, Bunglow No 14, VIP Road, Larkana, Sindh, Pakistan; 5 Incharge Leprosy Centre, Larkana, Sindh, Pakistan; 6 Department of Parasitology, Kochi Medical School, Kochi University, Oko-cho Kohasu, Nankoku-shi, Kochi 783-8505, Japan

## Abstract

The natural infection of phlebotomine sand flies by *Leishmania *parasites was surveyed in a desert area of Pakistan where cutaneous leishmaniasis is endemic. Out of 220 female sand flies dissected, one sand fly, *Phlebotomus kazeruni*, was positive for flagellates in the hindgut. Analyses of cytochrome *b *(*cyt *b), glycosomal glyceraldehyde phosphate dehydrogenase (*gGAPDH*) and small subunit ribosomal RNA (SSU rRNA) gene sequences identified the parasite as a *Trypanosoma* species of probably a reptile or amphibian. This is the first report of phlebotomine sand flies naturally infected with a *Trypanosoma *species in Pakistan. The possible infection of sand flies with *Trypanosoma *species should be taken into consideration in epidemiological studies of vector species in areas where leishmaniasis is endemic.

## Findings

Phlebotomine sand flies are blood-sucking insects belonging to the family Psychodidae in the order Diptera [1]. The identification of sand fly species is epidemiologically very important because less than 10 percent of over 800 species described are responsible for the transmission of human pathogens such as flagellate protozoa of the genus *Leishmania *[1-3]. Some sand fly species are reported to transmit non-pathogenic flagellates of *Endotrypanum *species, originally identified as intraerythrocytic parasites of sloths in the New World [4]. In addition, some *Trypanosoma* species of mammals, lizards, snakes and toads are transmitted by phlebotomine sand flies [5-11]. Since the flagellated forms of these parasites in the insect gut are morphologically similar to those of *Leishmania*, careful differentiation is needed for the epidemiological study of the vectors responsible for circulating *Leishmania *species.

In this study, the natural infection of sand flies by *Leishmania *was surveyed in a desert area of Pakistan where cutaneous leishmaniasis is endemic. Sand flies were captured at Sono Khan (26° 52'N, 68° 03'E), Sindh Province on the 28-29^th ^of June 2004. Shannon traps with a single compartment (1.0 × 1.0 × 1.3 meters in width, length and height, respectively) were used for the collection. The captured sand flies were dissected and the species identified based on the morphology of their spermathecae. These flies were also examined for *Leishmania *in the gut microscopically at × 400 magnification. The flagellates detected were inoculated into Difco blood agar (USMARU) biphasic medium containing 20% defibrinated rabbit blood, and later co-cultured with *Spodoptera frugiperda Sf*9 insect cells in Grace's medium supplemented with 10% fetal calf serum. The rest of the flagellates in the gut of the sand fly were fixed in absolute ethanol for molecular biological analyses. Genomic DNA was extracted from the ethanol-fixed specimen, and the cytochrome *b *(*cyt *b) gene was amplified from the flagellate with primers prepared for the leishmanial *cyt *b gene (L.cyt-S: GGTGTAGGTTTTAGTYTAGG and L.cyt-R: CTACAATAAACAAATCATAATATRCAATT) [12]. The glycosomal glyceraldehyde phosphate dehydrogenase (*gGAPDH*) and small subunit ribosomal RNA (SSU rRNA) genes were also amplified from the parasite using *gGAPDH* (G3: TTYGCCGYATYGGYCGCATGG and G5: ACMAGRTCCACCACRCGGTG) and SSU rRNA-specific primers (TRY927F: GAAACAAGAAACACGGGAG and TRY927R: CTACTGGGCAGCTTGGA) designed for trypanosomatids [13,14]. The PCR products were cloned into the pGEM-T Easy Vector (Promega, Madison, WI), and the sequences of the inserts of the plasmids were determined by the dideoxy chain termination method using a BigDye Terminator v3.1 Cycle Sequencing Kit (Applied Biosystems, Foster City, CA). The gGAPDH and SSU rRNA gene sequences were aligned with CLUSTAL W software [15] and examined using the program MEGA (Molecular Evolutionary Genetics Analysis) version 4.0 [16]. Phylogenetic analyses were performed by the neighbor-joining (NJ) and maximum parsimony (MP) methods with the distance algorisms available in the MEGA package [16].

**Figure 1:**
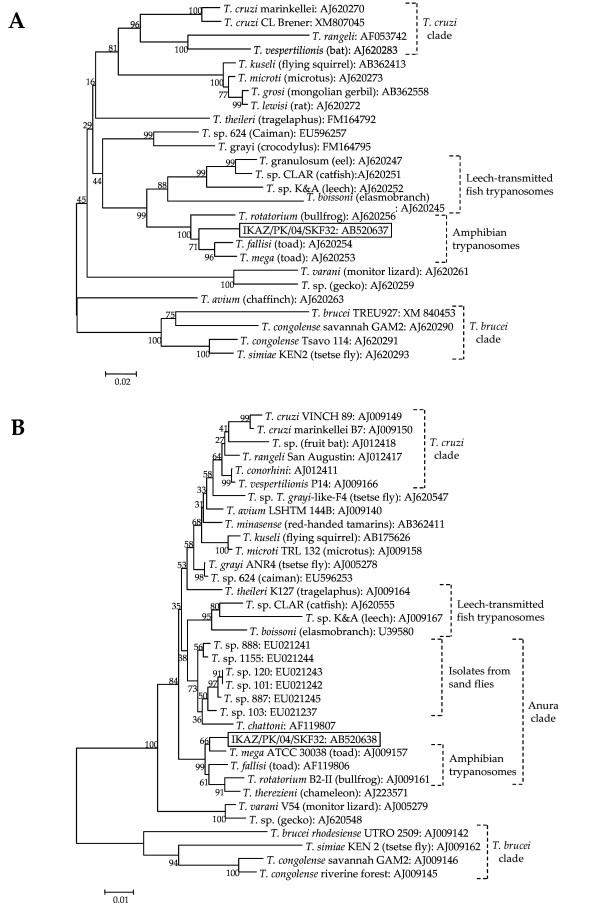
**Phylogenetic tree of *gGAPDH *(A) and SSU rRNA (B) gene sequences among species**. The *gGAPDH *and SSU rRNA genes were amplified from isolates of flagellates from *P. kazeruni *(IKAZ/PK/04/SKF32), and the sequences were determined. Phylogenetic analyses of *gGAPDH *and SSU rRNA gene sequences were performed by the neighbor-joining method together with those from 25 and 34 *Trypanosoma *species, respectively. The sequences from the database are represented by "the name of the species and isolate (host of the isolate): GenBank accession number". The scale bar represents 0.02 and 0.01% divergence, respectively. Bootstrap values are shown above or below branches.

**Figure 2:**
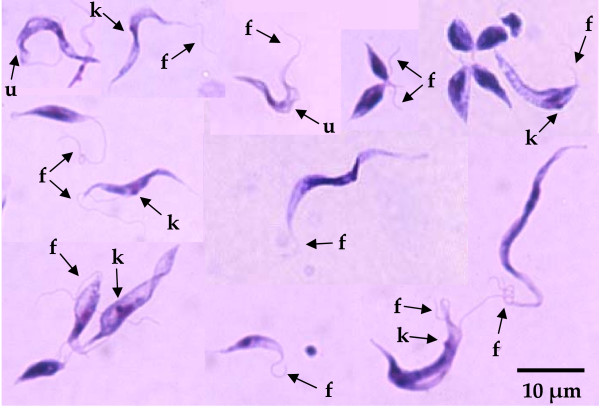
***Trypanosoma *species isolated from a phlebotomine sand fly *P. kazeruni***. Glass-slide smears were prepared from the culture in Grace's medium co-cultivated with *Sf*9 insect cells and stained with Giemsa staining. Rosettes of parasites with an oval shape and epimastigotes varying in morphology were observed. k, kinetoplast; f, flagellum; u, undulating membrane.

In this survey, 220 female sand flies were dissected for identification at the species level, and four species of the genus *Phlebotomus *were recognized. Among them, the two most prevalent species were identified as *Phlebotomus (P.) sergenti *(39 flies) and *P. papatasi *(27 flies). Other *Phlebotomus *species were *P. nuri *(10 flies) and *P. kazeruni *(1 fly), and the rest (143 flies) were identified as *Sergentomyia *species. The natural infection of sand flies by flagellates was detected in the hindgut of *P. kazeruni*. The *cyt *b gene sequence of the parasite was determined and analyzed with the BLASTn program. Unexpectedly, the sequence showed only 80.6-82.8% homology with those of *Leishmania *species and 83.6% homology with that of *Trypanosoma (T.) brucei brucei*, suggesting the flagellate belonged to the genus *Trypanosoma*. For further characterization, the *gGAPDH *and SSU rRNA genes were amplified from the flagellate and the sequences were analyzed since these genes have been well studied in trypanosomatids [13,14]. The *gGAPDH *and SSU rRNA genes from the parasite (DDBJ accession numbers: AB520637 and AB520638, respectively) had more than 90% sequence similarity with those of amphibian trypanosomes such as *T. fallisi, T. mega *and *T. rotatorium *isolated from toad and frog. Phylogenetic analyses of the *gGAPDH *and SSU rRNA genes showed that the flagellate within *P. kazeruni *belongs to a clade of amphibian trypanosomes that includes a chameleon trypanosome, *T. therezieni*, but the sequences did not completely match those from any reported species (Fig. 1A and 1B). In addition, the parasite from *P. kazeruni *located in a separate clade from *Trypanosoma *species isolated from Amazonian sand flies, which have closer relationships with anuran trypanosomes captured in Amazonia [10] (Fig. 1B). These results strongly suggested that the parasite is a novel or genetically uncharacterized *Trypanosoma *species of an amphibian or possibly reptile. The parasite was successfully isolated as a culture (code number: IKAZ/PK/04/SKF32), which was confirmed to correspond to the original by the SSU rRNA gene analysis. The parasites markedly grew in Grace's medium in a co-culture with *Sf*9 insect cells. On glass-slide smears prepared from the liquid culture, rosettes of parasites with an oval shape and epimastigotes varying in shape and length were observed (Fig. 2). The parasites showed the features of *Trypanosoma *species, demonstrating characteristics such as a flagellum, a kinetoplast and an undulating membrane (Fig. 2). The research area was a desert community, and no amphibian was observed during the field trip. However, numbers of geckoes were found on the walls of houses, including sites where the Shannon traps were set. Thus, two Japanese reptile species (*Takydromus tachydromoides *and *Gekko japonicus*) and one Japanese amphibian (*Hyla japonica*) were intraperitoneally injected with 5 × 10^6 ^parasites as a preliminary search for the vertebrate host of the parasite; however, no parasites were observed in peripheral blood 3 weeks after the inoculation. The possibility of mammalian hosts was also addressed since the frog trypanosome, *T. rotatorium*, has been reported to infect mice under experimental conditions [17]. The flagellate was inoculated intracutaneously into BALB/c mice and Mongolian gerbils (*Meriones unguiculatus*), but its DNA was not detected in peripheral blood and lymphoid tissues by PCR after 6 weeks. Based on the findings obtained by phylogenetic analyses and experimental infection, the parasite is presumably infectious to certain species of reptiles or amphibians in Pakistan. Further study is needed to identify the vertebrate host of the parasite species. The procedures were performed according to the recommendations of the ethics committee for animal experimentation, Faculty of Agriculture, Yamaguchi University.

In the present study, a *Trypanosoma *species was isolated from *P. kazeruni*, which is a xerophilic species distributing broadly throughout North Africa and West Asia with little-known behavior [18-20], and the parasite was genetically characterized. To date, trypanosomes in sand flies have been reported on the African and American continents [5-11], but this is the first record of phlebotomine sand flies naturally infected by a *Trypanosoma *species in Asia. Pakistan is a tropical and subtropical country located in the northwest of South Asia and has areas where leishmaniasis is highly endemic [21,22]. Although information on the endemicity and spread of cutaneous leishmaniasis is accumulating [23-26], the sand fly species responsible for the transmission is poorly understood. To date, no natural infections of sand flies by flagellates have been reported in Pakistan, and further efforts are ongoing on this matter. Thus, careful identification of the parasites is required for the epidemiological study of sand fly species responsible for the transmission of *Leishmania *protozoa.

## Competing interests

The authors declare that they have no competing interests.

## Authors' contributions

HK was fully involved in all phases of the study, including field research, sample collection, laboratory work, and writing the manuscript. HU and HS were involved in the molecular works. AMB, FRS and JHB organized and conducted field research. HU and YH conducted field research and sample collection. HK, HI and YH drafted the manuscript. YH supervised the overall conduct of the study. All authors read and approved the final manuscript.

## References

[B1] Munstermann LE Phlebotomine sand flies, the Psychodidae Biology of Disease Vectors 2004 Second Elsevier, San Diego CA 141 151

[B2] Desjeux P Leishmaniasis. Public health aspects and control Clin Dermatol 1996 14 417 423 10.1016/0738-081X(96)00057-0 8889319

[B3] Bates PA *Leishmania *sand fly interaction: progress and challenges Curr Opin Microbiol 2008 11 340 344 10.1016/j.mib.2008.06.003 18625337 PMC2675783

[B4] Shaw JJ A possible vector of *Endotrypanum schaudinni *of the sloth *Choloepus hoffmanni*, in Panama Nature 1964 201 417 418 10.1038/201417a0 14110021

[B5] Anderson JR Ayala SC Trypanosome transmitted by *Phlebotomus *: first report from the Americas Science 1968 161 1023 1025 10.1126/science.161.3845.1023 5671479

[B6] Ayala SC Trypanosomes in wild California sandflies, and extrinsic stages of *Trypanosoma bufophlebotomi* J Protozool 1971 18 433 436 5132317 10.1111/j.1550-7408.1971.tb03349.x

[B7] Ayala SC McKay JG *Trypanosoma gerrhonoti *n. sp., and extrinsic development of lizard trypanosomes in California sandflies J Protozool 1971 18 430 433 5132316 10.1111/j.1550-7408.1971.tb03348.x

[B8] Naiff RD Barrett TV Freitas RA Isolation of *Trypanosoma freitasi *(Kinetoplastida: Trypanosomatidae) from *Psychodopygus claustrei *(Diptera: Psychodidae) Mem Inst Oswaldo Cruz 1989 84 273 275 10.1590/S0074-02761989000200020 2635753

[B9] Viola LB Campaner M Takata CS Ferreira RC Rodrigues AC Freitas RA Duarte MR Grego KF Barrett TV Camargo EP Teixeira MM Phylogeny of snake trypanosomes inferred by SSU rDNA sequences, their possible transmission by phlebotomines, and taxonomic appraisal by molecular, cross-infection and morphological analysis Parasitology 2008 135 595 605 10.1017/S0031182008004253 18371240

[B10] Ferreira RC De Souza AA Freitas RA Campaner M Takata CS Barrett TV Shaw JJ Teixeira MM A phylogenetic lineage of closely related trypanosomes (Trypanosomatidae, Kinetoplastida) of anurans and sand flies (Psychodidae, Diptera) sharing the same ecotopes in brazilian amazonia J Eukaryot Microbiol 2008 55 427 435 10.1111/j.1550-7408.2008.00342.x 19017063

[B11] Lemos M Morais DH Carvalho VT D'Agosto M First record of *Trypanosoma chattoni *in Brazil and occurrence of other *Trypanosoma *species in Brazilian frogs (*Anura, Leptodactylidae*) J Parasitol 2008 94 148 151 10.1645/GE-1095.1 18372634

[B12] Kato H Cáceres AG Gomez EA Mimori T Uezato H Marco JD Barroso PA Iwata H Hashiguchi Y Molecular mass screening to incriminate sand fly vectors of Andean-type cutaneous leishmaniasis in Ecuador and Peru Am J Trop Med Hyg 2008 79 719 721 18981511

[B13] Noyes HA Stevens JR Teixeira M Phelan J Holz P A nested PCR for the ssrRNA gene detects *Trypanosoma binneyi *in the platypus and *Trypanosoma *sp. in wombats and kangaroos in Australia Int J Parasitol 1999 29 331 339 10.1016/S0020-7519(98)00167-2 10221634

[B14] Hamilton PB Stevens JR Gaunt MW Gidley J Gibson WC Trypanosomes are monophyletic: evidence from genes for glyceraldehyde phosphate dehydrogenase and small subunit ribosomal RNA Int J Parasitol 2004 34 1393 1404 10.1016/j.ijpara.2004.08.011 15542100

[B15] Thompson JD Higgins DG Gibson TJ CLUSTAL W: improving the sensitivity of progressive multiple sequence alignment through sequence weighting, position-specific gap penalties and weight matrix choice Nucleic Acids Res 1994 22 4673 4680 10.1093/nar/22.22.4673 7984417 PMC308517

[B16] Tamura K Dudley J Nei M Kumar S MEGA4: Molecular Evolutionary Genetics Analysis (MEGA) software version 4.0 Mol Biol Evol 2007 24 1596 1599 10.1093/molbev/msm092 17488738

[B17] Hysek J Zizka Z Transmission of *Trypanosoma rotatorium *from frogs to white mice Nature 1976 260 608 609 10.1038/260608a0 1264222

[B18] Kamhawi S Abdel-Hafez SK Molyneux DH The behaviour and dispersal of sandflies in Ras el Naqb, south Jordan with particular emphasis on *Phlebotomus kazeruni* Parassitologia 1991 33 307 314 1841222

[B19] Hanafi HA Kanour WW Jr Beavers GM Tetreault GE Colonization and bionomics of the sandfly *Phlebotomus kazeruni *from Sinai, Egypt Med Vet Entomol 1999 13 295 298 10.1046/j.1365-2915.1999.00172.x 10514056

[B20] Toprak S Ozer N Sand fly species of Sanliurfa province in Turkey Med Vet Entomol 2005 19 107 110 10.1111/j.0269-283X.2005.00545.x 15752185

[B21] Khan SJ Muneeb S Cutaneous leishmaniasis in Pakistan Dermatol Online J 2005 11 4 15748545

[B22] Katakura K Molecular epidemiology of leishmaniasis in Asia (focus on cutaneous infections) Curr Opin Infect Dis 2009 22 126 130 10.1097/QCO.0b013e3283229ff2 19276879

[B23] Marco JD Bhutto AM Soomro FR Baloch JH Barroso PA Kato H Uezato H Katakura K Korenaga M Nonaka S Hashiguchi Y Multilocus enzyme electrophoresis and cytochrome *b *gene sequencing-based identification of *Leishmania *isolates from different foci of cutaneous leishmaniasis in Pakistan Am J Trop Med Hyg 2006 75 261 266 16896129

[B24] Noyes HA Reyburn H Bailey JW Smith D A nested-PCR-based schizodeme method for identifying *Leishmania *kinetoplast minicircle classes directly from clinical samples and its application to the study of the epidemiology of *Leishmania tropica *in Pakistan J Clin Microbiol 1998 36 2877 2881 9738037 10.1128/jcm.36.10.2877-2881.1998 PMC105081

[B25] Bhutto AM Soomro RA Nonaka S Hashiguchi Y Detection of new endemic areas of cutaneous leishmaniasis in Pakistan: a 6-year study Int J Dermatol 2003 42 543 548 10.1046/j.1365-4362.2003.01818.x 12839604

[B26] Myint CK Asato Y Yamamoto Y Kato H Bhutto AM Soomro FR Memon MZ Matsumoto J Marco JD Oshiro M Katakura K Hashiguchi Y Uezato H Polymorphisms of cytochrome *b *gene in *Leishmania *parasites and their relation to types of cutaneous leishmaniasis lesions in Pakistan J Dermatol 2008 35 76 85 10.1111/j.1346-8138.2008.00419.x 18271802

